# Identifying appropriate outcomes to help evaluate the impact of the *Canadian Guideline for Safe and Effective Use of Opioids for Non-Cancer Pain*

**DOI:** 10.1186/s12871-020-0930-4

**Published:** 2020-01-07

**Authors:** Michael Allen, Beth Sproule, Peter MacDougall, Andrea Furlan, Laura Murphy, Victoria Borg Debono, Norman Buckley

**Affiliations:** 1 Continuing Professional Development, Dalhousie University, Halifax, Canada; 2 Centre for Addiction & Mental Health (CAMH), Toronto, Canada; 3 Leslie Dan Faculty of Pharmacy, University of Toronto, Toronto, Canada; 4 Department of Anesthesia, Pain Management and Perioperative Medicine, Dalhousie University, Halifax, Canada; 5 Institute for Work & Health, Toronto, Canada; 6 Toronto Rehabilitation Institute, University Health Network, Toronto, Canada; 7 Michael G. DeGroote National Pain Centre, McMaster University, Hamilton, Canada

**Keywords:** Outcomes to evaluate guideline impact, Modified Delphi process, National Pain Centre, Chronic non-Cancer pain, Opioids

## Abstract

**Background:**

The *Canadian Guideline for Safe and Effective Use of Opioids for Chronic Non-Cancer Pain* (COG) was developed in response to increasing rates of opioid-related hospital visits and deaths in Canada, and uncertain benefits of opioids for chronic non-cancer pain (CNCP). Following publication, we developed a list of evaluable outcomes to assess the impact of this guideline on practice and patient outcomes*.*

**Methods:**

A working group at the National Pain Centre at McMaster University used a modified Delphi process to construct a list of clinical and patient outcomes important in assessing the uptake and application of the COG. An advisory group then reviewed this list to determine the relevance and feasibility of each outcome, and identified potential data sources. This feedback was reviewed by the National Faculty for the Guideline, and a National Advisory Group that included the creators of the COG, resulting in the final list of 5 priority outcomes.

**Results:**

Five outcomes were judged clinically important and feasible to measure: 1) Effects of opioids for CNCP on quality of life, 2) Assessment of patient’s risk of addiction before starting opioid therapy, 3) Monitoring patients on opioid therapy for aberrant drug-related behaviour, 4) Mortality rates associated with prescription opioid overdose and 5) Use of treatment agreements with patients before initiating opioid therapy for CNCP. Data sources for these outcomes included patient’s medical charts, e-Opioid Manager, prescription monitoring programs and administrative databases.

**Conclusion:**

Measuring the impact of best practice guidelines is infrequently done. Future research should consider capturing the five outcomes identified in this study to evaluate the impact of the COG in promoting evidence-based use of opioids for CNCP.

## Background

The *Canadian Guideline for Safe and Effective Use of Opioids for Chronic Non-Cancer Pain* [1] (COG) was initially released in April 2010 and an updated and revised version was published in 2017 [2]. This guideline was developed in response to the concern that Canada is the second highest user of opioids per capita in the world and that the rates of opioid prescribing and opioid-related hospital visits and deaths have been increasing [2]. The COG is officially endorsed by the Federation of Medical Regulatory Authorities of Canada (FMRAC) and all provincial medical colleges, and is freely available from the website of the Michael G. DeGroote National Pain Centre (NPC), McMaster University [3] and in a multi-layered and interactive format through MAGICapp [4]. Development of the 2010 COG was undertaken by the National Opioid Use Guideline Group (NOUGG) [5] working under the auspices of FMRAC [6]. Copyright was then transferred to the NPC which assumed responsibility for dissemination and any future update. Its update was then undertaken by the NPC at McMaster University with funding by Health Canada [7]. The NOUGG established a National Faculty that still exists today and whose members have had input into the COG. The National Faculty consists of professionals with expertise in pain management, addiction medicine, primary care, knowledge translation, and epidemiology and patient advocates and representatives, who are further divided into working groups of experts to ensure the ongoing dissemination, evaluation and revision of the COG. One of the working groups, the Guideline Impact Evaluation Working Group (EWG) was established to evaluate of the impact of the COG in Canada. This included establishing important outcomes to measure and determining where and how to obtain necessary data for guideline evaluation.

In this paper, we report the process used to identify key priority outcomes, and data sources for their measurement, for evaluation of the impact of the COG. The primary objective of this study was to identify those key priority outcomes and their associated measures for future evaluation. A cross-country collaboration is needed to evaluate the issue of opioid addiction and diversion in Canada and assess the COG’s impact on practice and patient outcomes. Thus, it is expected that the resulting outcomes and associated measures from this research reported in this paper will provide direction to the evaluation process.

## Methods

We used Moore’s model for evaluating educational interventions and their outcomes to identify priority outcomes and their associated measures for evaluation [8]. In Moore’s expanded outcomes model of learning outcomes for assessing educational interventions activities, we considered outcomes at the performance level (level 5) or higher. Level 5 evaluates how well participants demonstrate or do what the educational activity intended for them to do in their practice. The next two levels, patient health (level 6) and community health (level 7), evaluate the degree to which the health status of patients improves due to changes in practice behaviour of clinicians and the health status of a community of patients changes due to changes in practice behaviour [8].

**Fig. 1:**
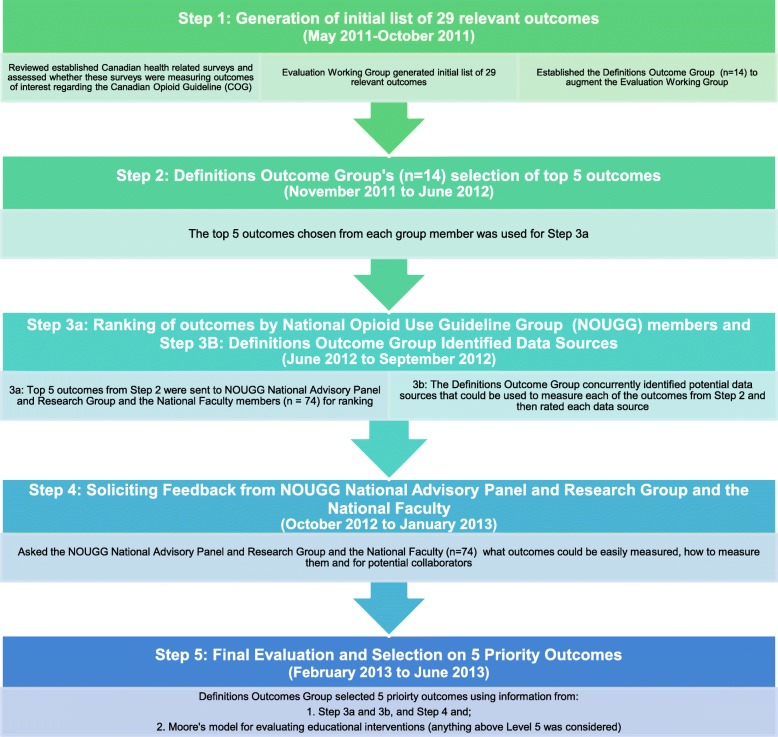
Research Outcomes Selection Process

The process to select outcomes took place over the period following the introduction of the 2010 COG from May 2011 through mid-2013 using a modified Delphi process [9, 10]. Figure 1 outlines the steps taken to select 5 priority outcomes. For Step 1, the EWG consulted with the Canadian Hypertension Education Program (CHEP), now known as Hypertension Canada, which has extensive experience in guideline evaluation [11, 12]. Based on Hypertension Canada’s experience, we reviewed established Canadian health related surveys to determine if they included outcome measures relevant to our objective. We found that the outcome measures from these surveys were not applicable to evaluating the COG and, as such, the EWG generated an initial list of 29 relevant outcomes to evaluate the impact of the 2010 COG and its 24 practice recommendations [13]. A Definitions Outcomes Group consisting of 14 physicians, pharmacists, and methodologists, with expertise in pain and addictions management, knowledge translation, and epidemiology categorized these outcomes as either practice or clinical outcomes, and further subdivided them into pain and addiction categories (Table 1). Reponses and data for Steps 2, 3 and 4 were collected using Opinio, online-based survey software [14]. None of the responses could be linked to an individual. For Step 2, each member of the Definitions Outcomes Group selected their top five choices. Outcomes that each member of the Definitions Outcomes Group chose as their top five were selected and used for Step 3a.

**Table 1:** Initially generated 29 outcomes by the Definitions Outcomes Group (*n* = 14)

Practice Outcomes	
Pain	Addictions
1. Assessment of pain using a validated pain scale or another validated method 2. Assess function with scale – intended as part of quality of life assessment 3. Prescribing opioids to patients for conditions for which evidence has shown opioids to be effective for management of CNCP 4. Prescribing of opioids at or greater than the watchful dose (200 mg of morphine equivalent per day) for CNCP 5. Discontinuation or tapering in patients experiencing adverse effects or insufficient opioid effectiveness 6. Referral of patients with CNCP to a pain specialist or pain centre 7. Safe initiation of fentanyl during an opioid trial using the “stepped approach” for CNCP 8. Use of meperidine and pentazocine for treating CNCP 9. Number and proportion of CNCP patients who receive non-drug treatments 10. Concomitant prescribing of benzodiazepines and opioids	1. Assessment of patient’s risk of addiction before starting opioid therapy by prescribers, such as use of tools. 2. Ordering of urine drug screening for patients before starting opioids and when monitoring the patient 3. Use of treatment agreements with patients before initiating opioid therapy for CNCP 4. Use of patient information from prescription monitoring programs to monitor patients on opioid therapy for aberrant drug-related behaviours, where available. 5. Clinician response to the detection of aberrant drug-related behaviours in patients on opioid therapy. 6. Referrals to addictions services 7. Methadone/buprenorphine prescribing 8. Monitoring patients on opioid therapy for aberrant drug-related behaviours 9. Acute and urgent health-care facilities’ use of policies to provide guidance on opioid prescribing
Clinical Outcomes	
Pain	Addictions
1. Amount of weak and strong opioids prescribed by jurisdiction and per patient with CNCP 2. Types and amounts of adjunctive medications prescribed for CNCP patients taking opioids 3. Suicide rates associated with inadequately controlled pain 4. Emergency room visit rates associated with inadequate pain control 5. Effects of CNCP and taking opioids for CNCP on quality of life	1. Prevalence and incidence of prescription opioid addiction 2. Mortality rates associated with prescription opioid overdose 3. Emergency room visit rates associated with prescription opioid overdose 4. Proportion of patients on opioid therapy for CNCP who exhibit aberrant drug-taking behavior 5. Extent of prescription opioid diversion

Chronic Non-Cancer Pain (CNCP)

For Step 3a, the outcomes that each member of the Definitions Outcomes Group chose as their top five (from Step 2) were then sent to the original NOUGG National Advisory Panel, Research Group, and the National Faculty (*n* = 74) who were asked to rank their top five outcomes from 1 (first choice) to 5 (fifth choice). The National Faculty, in addition to including professionals with expertise in pain management, addiction medicine, primary care, knowledge translation, and epidemiology, also includes patient advocates and representatives. We developed weighted scores for each outcome as follows: A first choice (ranking of 1) was assigned 5 points; a second choice (ranking of 2) was assigned 4 points; a third choice (ranking of 3) was assigned 3 points; a fourth choice (ranking of 4) was assigned 2 points; and a fifth choice (ranking of 5) was assigned 1 point. These points were multiplied by the number of respondents giving that ranking and the points summed for a weighted score. For example, an outcome ranked first by 11 people, second by 7 people, third by 8 people, fourth by 1 person, and fifth by 5 people received a weighted score of 114 [(5 points × 11 people) + (4 points × 7 people) + (3 points × 8 people) + (2 points × 1 person) + (1 point × 5 people) = 114].

For Step 3b, the Definitions Outcome Group identified potential data sources to measure each of the outcomes from Step 2. This was done concurrently with Step 3a. Each data source was rated on a 5-point scale (a point of 1 meant “least” and a point of 5 meant “most”) based on the following criteria adapted from the Nova Scotia Department of Health and Wellness, Hypertension Indicator Priority Setting Tool [15]:

- *Feasibility* - Data to measure outcome should be easily accessible;
- *Credibility* - Should be valid and reliable;
- *Comparability* - Could be used to compare across geographic areas and across time; and
- *Understandability* - Should be easy to interpret with no ambiguity as to whether performance has improved or deteriorated.

For Step 4, the list of outcomes determined at Step 2 and potential data sources from Step 3b were sent again to the NOUGG National Advisory Panel and Research Group and the National Faculty (*n* = 74) soliciting their feedback on which outcomes they thought could be easily measured, and how, In addition, they were asked to identify potential collaborators to assess the outcomes that will generated from this study to help evaluate the impact of the COG on practice.

For Step 5, in a face-to face discussion the Definitions Outcome Group agreed to select 5 outcomes as priority for the evaluation of the impact of the COG, based on the following information in its totality:

1. The weighted scores and associated rank of the outcomes from Step 3a;
2. The evaluation of the potential data sources for the outcomes based on feasibility, credibility, comparability and understandability from Step 3b;
3. Solicited feedback from Step 4; and
4. The level the outcome meets on the Moore’s model for evaluating educational interventions.

Any outcome at or above the performance level (Level 5) of the Moore’s model for evaluating educational interventions deemed by the Definitions Outcome Group was considered. We think it necessary to aim for a conceptually high level on the Moore’s model when evaluating a national clinical guideline as outcomes at or above Level 5 are better able to indicate an impact on clinical practice and patient outcomes.

## Results

For Step 1, the EWG generated an initial list of 29 outcomes to evaluate the impact of the 2010 COG and its 24 practice recommendations [13] (Table 1). For Step 2, 16 of the 29 outcomes were selected by at least one Definitions Outcomes Group member as their top five (Table 2). Table 3 highlights the weighted scores and the rankings from Step 3a. Forty-five out of 74 people responded to this survey for a response rate 60.8%. Weighted scores ranged from 14 to 146. Table 4 highlights the data sources identified to measure each of the 16 outcomes from Step 2 and the ratings for each data source on a 5-point scale, based on feasibility, credibility, comparability and understandability. For Step 4, seven out of 74 people responded and they suggested if possible, to not rely on self-reported data and identified potential collaborators such as Workers Compensation Boards which have records on medication prescriptions for clients, Prescription Monitoring Programs, and the Centre for Effective Practice [16]. It was also mentioned that there is a point of care tool for prescribing opioids called the Opioid Manager [6] which is available in an electronic format and amenable to data collection.

**Table 2:** Top 16 Outcomes selected by the Evaluation Working Group (*n* = 14)

1. Amount of weak and strong opioids prescribed by jurisdiction and per patient with CNCP 2. Assessment of pain using a validated pain scale or another validated method 3. Discontinuation or tapering in patients experiencing adverse effects or insufficient opioid effectiveness 4. Concomitant prescribing of benzodiazepines and opioids 5. Prevalence and incidence of prescription opioid addiction 6. Emergency room visit rates associated with prescription opioid overdose 7. Assessment of patient’s risk of addiction before starting opioid therapy by prescribers, such as use of tools. 8. Use of treatment agreements with patients before initiating opioid therapy for CNCP 9. Monitoring patients on opioid therapy for aberrant drug-related behaviour 10. Use of patient information from prescription monitoring programs to monitor patients on opioid therapy for aberrant drug-related behaviours, where available 11. Acute and urgent health-care facilities’ use of policies to provide guidance on opioid prescribing 12. Safe initiation of fentanyl during an opioid trial using the “stepped approach” for CNCP 13. Mortality rates associated with prescription opioid overdose 14. Types and amounts of adjunctive medications prescribed for CNCP patients taking opioids 15. Effects of CNCP and taking opioids for CNCP on quality of life 16. Prescribing of opioids at or greater than the watchful dose (200 mg of morphine equivalent per day) for CNCP	

Chronic Non-Cancer Pain (CNCP)

**Table 3:** Weighted Score and Ranking of Outcome by NOUGG Members and the National Faculty (*n* = 45)

Rank	Outcome	Weighted Score
1	Effects of CNCP and taking opioids for CNCP on quality of life	146
2	Assessment of patient’s risk of addiction before starting opioid therapy by prescribers, such as use of tools.	138
3	Monitoring patients on opioid therapy for aberrant drug-related behaviour	78
4	Prescribing of opioids at or greater than the watchful dose (200 mg of morphine equivalent per day) for CNCP	74
5	Mortality rates associated with prescription opioid overdose	64
6	Prevalence and incidence of prescription opioid addiction	62
7	Discontinuation or tapering of opioids who experience adverse effects or insufficient opioid effectiveness	59
8	Assessment of pain using a validated pain scale or another validated method	44
9	Use of treatment agreements with patients before initiating opioid therapy for CNCP	43
10	Use of patient information from prescription monitoring programs to monitor patients on opioid therapy for aberrant drug-related behaviours, where available	42
11	Emergency room visit rates associated with prescription opioid overdose	30
12	Concomitant prescribing of benzodiazepines and opioids	29
13	Amount of weak and strong opioids prescribed by jurisdiction and per patient with CNCP	29
14	Acute and urgent health-care facilities’ use of policies to provide guidance on opioid prescribing	25
15	Types and amounts of adjunctive medications prescribed for CNCP patients taking opioids	23
16	Safe initiation of fentanyl during an opioid trial using the “stepped approach” for CNCP	14

Chronic Non-Cancer Pain (CNCP), National Opioid Use Guideline Group (NOUGG)

**Table 4:** Potential data sources assessed for feasibility, credibility, comparability and understandability on a 5-point scale (1 to 5)

Outcome	Potential Data Source	Feasible*^a^	Credible*^b^	Comparable*^c^	Understandable*^d^
1. Effects of CNCP and taking opioids for CNCP on quality of life	Chart review	3	3	3	4
	Self-report	5	4	4	5
2. Assessment of patient’s risk of addiction before starting opioid therapy by prescribers, such as use of tools.	Chart review	2	4	5	4
	Self-report	5	2	5	4
3. Monitoring patients on opioid therapy for aberrant drug-related behaviour	Chart review	1	2	2	2
	Self-report	4	2	3	3
4. Prescribing of opioids at or greater than the watchful dose (200 mg of morphine equivalent per day) for CNCP	Administrative database	5	5	5	5
5. Mortality rates associated with prescription opioid overdose	Administrative database	5	4	4	5
6. Prevalence and incidence of prescription opioid addiction	Chart review	1	2	2	2
	Self-report	5	2	5	5
	Administrative database	2	3	3	4
7. Discontinuation or tapering in patients experiencing adverse effects or insufficient opioid effectiveness	Chart review	1	2	2	2
	Self-report	4	2	3	3
	Administrative database	4	3	2	4
8. Assessment of pain using a validated pain scale or another validated method	Chart review	2	4	5	4
	Self-report	5	2	5	4
9. Use of treatment agreements with patients before starting opioid therapy for CNCP	Chart review	2	4	5	4
	Self -report	5	2	5	4
10. Use of patient information from prescription monitoring programs to monitor patients on opioid therapy for aberrant drug-related behaviours, where available	Prescription monitoring program(s)	5	5	3	5
11. Emergency room visit rates associated with prescription opioid overdose	Administrative database	5	4	4	5
12. Concomitant prescribing of benzodiazepines and opioids	Chart review	2	4	5	5
	Self-report	5	2	5	5
	Administrative database	4	4	5	5
13. Amount of weak and strong opioids prescribed by jurisdiction and per patient with CNCP	Chart review	4	4	4	5
	Self-report	5	3	4	5
	Administrative database	5	5	4	5
14. Acute and urgent health-care facilities’ use of policies to provide guidance on opioid prescribing	Facilities’ policies	4	4	5	5
15. Types and amounts of adjunctive medications prescribed for CNCP patients taking opioids	Chart review	3	4	4	5
	Self-report	5	3	4	5
	Administrative database	3	3	3	5
16. Safe initiation of fentanyl during an opioid trial using the “stepped approach” for CNCP	Chart review	2	4	5	5
	Self-report	5	2	5	5
	Administrative database	4	4	5	5

Chronic Non-Cancer Pain (CNCP)

*Scale from 1 to 5 where a point of 1 means “least” and a point of 5 means “most”

^a^Adapted study definition: Data to measure outcome should be easily accessible

^b^Adapted study definition: Should be valid and reliable,

^c^Adapted study definition: Could be used to compare across geographic areas and across time,

^d^Adapted study definition: Should be easy to interpret with no ambiguity as to whether performance has improved or deteriorated

For Step 5, the Definitions Outcome Group selected the following five outcomes (See Table 5) as priority for the evaluation of the impact of the COG on practice and patient outcomes:

1. Effects of CNCP and taking opioids for CNCP on quality of life
2. Assessment of patient’s risk of addiction before starting opioid therapy by prescribers, such as the use of standardized tools.
3. Monitoring patients on opioid therapy for aberrant drug-related behaviours
4. Mortality rates associated with prescription opioid overdose
5. Use of treatment agreements with patients before initiating opioid therapy for CNCP

**Table 5:** Discussion points by the Evaluation Working Group on the final five outcomes for guideline evaluation

Outcome	Measurements	Sources of data	Level on Moore Hierarchy	Rank^a^
1. Effects of CNCP and taking opioids for CNCP on quality of life	1. Scores on validated quality of life and function scales for patients with CNCP (e.g. SF-36^b^) 2. Change in scores on validated quality of life scales and function scales for patients with CNCP treated with opioids (e.g. SF-36^b^)	Opioid Manager Chart review	Level 6. Degree of improvement of patient health status due to changes in practice behaviour	1
2. Assessment of patient’s risk of addiction before starting opioid therapy by prescribers, such as the use of tools.	1. Proportion of patients who have their risk for addiction assessed with a screening tool prior to initiating opioid therapy for CNCP. 2. Frequency of methods used by health care providers to assess risk of addiction, e.g., validated scales vs informal assessment	Opioid Manager Chart review	Level 5. Clinicians’ application of knowledge in practice setting	2
3. Monitoring patients on opioid therapy for aberrant drug-related behaviour	1. Proportion of patients on opioid therapy for CNCP who are monitored for aberrant drug-related behaviours by physicians and pharmacists 2. Proportion of physicians and pharmacists who routinely monitor for aberrant drug-related behaviour in their patients on opioid therapy for CNCP 3. Proportion of physicians and pharmacists who routinely monitor for aberrant drug-related behaviour in their patients on opioid therapy using prescription monitoring program patients information, where available.	Opioid Manager Prescription monitoring program inquiries Chart review	Level 5. Clinicians’ application of knowledge in practice setting	3
4. Mortality rates associated with prescription opioid overdose	1. Number of people whose deaths were associated with prescription opioid overdose by year and by region	Administrative databases (e.g. Coroner’s data^b^) Electronic Health Records	Level 7. Degree of improvement of a community of patients due to changes in practice behaviour	5
5. Use of treatment agreements with patients before initiating opioid therapy for CNCP	1. Proportion of patients who have a treatment agreement with their physician prior to starting opioid therapy. 2. Proportion of physicians who employ a treatment agreement prior to initiating opioid therapy for CNCP	Opioid Manager Chart review	Level 5. Clinicians’ application of knowledge in practice setting	9

Chronic Non-Cancer Pain (CNCP)

^a^The rank comes from Step 3a based on the weighted scores. For example, Outcome 1 has a rank of 1 meaning that it had the highest weighted score from Step 3a. These weighted scores and associated rankings can be seen in Table 3

^b^Just an example provided; not identified as a result of outcome selection process

Table 5 summarizes the main discussion points of the Definitions Outcome Group when they assessed and chose these five priority outcomes to help evaluate the impact of the COG on practice and patient care; including the associated measures and data sources that could be used, the ranking they received from NOUGG members and the National Faculty from Table 3 and the level the outcome was on the Moore’s model [8].

## Discussion

### Key findings

Our study identified five priority outcomes, and a series of possible associated measures and data sources, to evaluate the impact of the COG. Data to assess four of the five identified priority outcomes (Outcomes 1, 2, 3 and 5) would be best collected directly by using electronic patient health records, and integrated electronic practice tools, such as the Opioid Manager. The Opioid Manager is both a tool and source of data specifically developed to monitor patients with CNCP and on opioids, and captures these outcomes [6, 17, 18]. Medical charts may be another source of data for these outcomes. For Outcome 1, regarding the effects of CNCP and taking opioids for CNCP on quality of life, the Short Form-36 (SF-36) is an example of a validated and reliable measure to assess a patient’s quality of life [19] and can be used in parallel with the Opioid Manager. This example measure, the SF-36, was not identified as a result of this study’s process for selecting outcomes but rather is provided as good example of a measure that can be used for Outcome 1. For Outcome 3, monitoring patients on opioid therapy for aberrant drug-related behaviour, one could also utilize prescription monitoring programs. Outcome 4, *mortality rates associated with prescription opioid overdose*, could be informed through administrative databases (e.g. coroner’s data and electronic health records). However, further examination of Outcome 4 is warranted as an opioid overdose can involve multiple types of drugs, such as more than one opioid (e.g. fentanyl and an opiate alkaloid), and alcohol. Thus, assessment of mortality rates associated with opioids requires concurrent study of a person’s opioid and other medication use history prior to an opioid overdose [20]. This includes the characterization of prescribed opioids deemed 1) responsible for the overdose, 2) contributing to the overdose (e.g. multiple opioid types or Central Nervous System depressants identified from coroner’s report), or 3) influential in a patient’s history relating to their overdose (e.g. prescribed in their past), and assessment of their concomitant medications. As mentioned above, this characterization can also be found in a coroner’s report and in a patient’s electronic health records.

### Explanation of the findings

We believe this research provides insight into a systematic approach that was and can be used again in the future to validate outcomes for guideline evaluation and evaluation processes for other opioid guidelines and perhaps for other guidelines in general. We undertook this systematic approach with the intent to assess the impact of the COG on practice and patient care. The COG provides a knowledge base which can support consistent education and outcome measures. It is helpful to consider our five priority outcomes in relation to Moore’s expanded outcomes model of learning outcomes for assessing education interventions [8]; one of the outcomes (Outcome 4) is at Level 7 (community health outcome), Outcome 1 is at Level 6 (Individual patient health outcomes), and Outcomes 2, 3, and 5, are at Level 5 (Clinicians’ application of knowledge in practice setting or competency).

Currently, there is a lack of studies evaluating the impact of the COG and medical guidelines in general on clinical practice and patient outcomes in Canada [21, 22]. In relation to other efforts and studies to evaluate the impact of guidelines or recommendations on clinical practice, much work has been done by Hypertension Canada to monitor the impact of such efforts to deal with hypertension in Canada in addition to an implementation (knowledge translation) program for such guidelines into primary care [11, 23]. Their initial key outcome was to improve the treatment and control of hypertension in Canada by creating and implementing recommendations. In 2003, they then set up a task force of experts in the field to assess their efforts to improve the treatment and control of hypertension [11]. Their task force, similar to ours, identified a number of areas or outcomes for surveillance in relation to hypertension. They used nationally administered surveys through Statistics Canada as a tool for surveillance. In the future, it may be possible for us to also work with Statistics Canada to include questions addressing outcome 1, on the effects of CNCP and taking opioids for CNCP on quality of life and other outcomes where feasible.

### Limitations

There is little in the way of formal guidance available in evaluating the impact of clinical practice guidelines. Thus, the working group found it necessary to seek guidance from several sources, and convene several groups of advisors, in order to assist in the de novo development of outcomes relevant to the clinical areas addressed by the guideline. We believe that this potential limitation was mitigated by the strength and breadth of expertise of the advisors involved. For instance, the Evaluation Working Group, and the National Pain Centre, which is the sponsoring body for the National Faculty, includes practitioners in the fields of pain and addiction, research scientists, clinicians, pharmacists, physicians and nurses for multi-disciplinary input. The Definitions Outcome Group included scientists with experience in population health research who informed the feasibility of proposed measures, and individuals who had published in support of greater restriction of opioid use as well as greater availability of opioids for analgesia, representing the breadth of clinical problems faced. Additionally, the 5 priority outcomes generated from this process were determined after introduction of 2010 COG but prior to the 2017 update. These priority outcomes are still relevant to assess as most still relate to the updated guideline recommendations and are based on upon a rigorous process that solicited the view of professionals with expertise in pain management, addiction, knowledge translation, epidemiology and patient advocacy, who deemed these outcomes worth evaluating in regards to CNCP management and proper opioid use. However, it is important to note that both the 2010 COG [13] and the most recent 2017 COG [4] were unable to make a strong recommendation or provide strong evidence respectively about the use of treatment agreements, even though it was found in our study that such an outcome would be of importance to evaluate. This suggests it is very likely, in the future, that this outcome would still come up as something important to evaluate as it did in our study despite both the 2010 and 2017 COG stating that such a tool may be helpful to use. This outcome was evidently in the minds of who were surveyed in this study and the modified Delphi process we undertook drew out this outcome.

## Conclusion

We identified five priority outcomes, and a series of possible associated measures and data sources to target the evaluation of the guideline’s impact on practice and patient care. Our goal is to make these five priority outcomes widely known to investigators and organizations for consideration in the design of future research studies or funding calls. We welcome collaboration from other groups and individuals as we move forward on evaluating the five priority outcomes we have identified. We also welcome input from and collaboration with groups that may have practical ideas about measuring other outcomes, or have existing data that could contribute to the COG’s evaluation. Our approach is to be collaborative and comprehensive with evaluating the impact of COG on practice.

## Data Availability

Not applicable.

## Abbreviations

CHEP: Canadian Hypertension Education Program
COG: Canadian Guideline for Safe and Effective Use of Opioids for Chronic Non-Cancer Pain
EWG: Evaluation Working Group
FMRAC: Federation of Medical Regulatory Authourities of Canada
NOUGG: Opioid Use Guideline Group
NPC: National Pain Centre
